# Addressing financial biases in university undergraduates: Unveiling connections with knowledge, behaviours and attitudes

**DOI:** 10.1371/journal.pone.0336274

**Published:** 2025-11-06

**Authors:** Celia López-Penabad, Marcos Álvarez-Espiño, Leandro Benito-Torres

**Affiliations:** 1 Department of Financial Economics and Accounting, ECOBAS, Universidade de Santiago de Compostela, Spain; 2 Department of Organisation of Companies and Commercialisation, Universidade de Santiago de Compostela, Spain; Academia de Studii Economice din Bucuresti, ROMANIA

## Abstract

**Purpose:**

This study investigates how financial knowledge, behaviours, and attitudes shape the prevalence of financial biases among Economics students at the University of Santiago de Compostela.

**Methodology:**

Based on survey data from 403 first- and fourth-year students, a composite bias index—covering overconfidence, gambler’s fallacy, and herd behaviour—is constructed using the Benefit of the Doubt method. Truncated regressions explore the influence of financial capability.

**Findings:**

Results show attitudinal factors explain biases better than knowledge. Surprisingly, behaviours such as long-term planning and fraud avoidance increase susceptibility to biases. These findings highlight the complexity of financial decision-making and the need for emotionally aware, bias-targeted financial education.

**Value:**

This paper introduces a novel approach by constructing multiple financial bias indices and calling for hands-on, behaviour-focused financial education.

## 1. Introduction

Classical economic theory, particularly the orthodox paradigm of perfect rationality, assumes that individuals make financial decisions as if they were highly efficient algorithms, guided solely by the rational pursuit of expected utility [1,2]. This implies that individuals can assess all possible financial choices and their consequences under conditions of absolute certainty, ultimately selecting the most optimal course of action. However, empirical evidence challenges this notion, as financial markets frequently show speculative bubbles, crashes, and herd-driven fluctuations that do not align with the fully rational *homo economicus* paradigm [1,2].

These deviations from rationality can be explained by the theory of bounded rationality [2,3], which posits that human decision-making is constrained by cognitive limitations, information asymmetries, and time restrictions [3,4]. In such scenarios, particularly in financial decision-making, individuals rely on heuristics, emotions, expectations, and social influences to “simplify” complex choices [2,5]. While heuristics can be efficient shortcuts, they also introduce cognitive biases that may distort judgment and lead to suboptimal financial behaviours [3,5]. Keynes [6] described these irrational tendencies as animal spirits, which influence economic behaviour beyond purely rational calculations.

In this context, financial decision-making requires capabilities, or the necessary tools to effectively manage available resources [7], whether related to time, money or knowledge. A higher level of financial literacy is associated with a greater ability to analyse key elements of a decision [7], reducing reliance on heuristics and mitigating the influence of behavioural biases.

Financial literacy has been broadly defined as the ability to manage assets and financial resources to enhance financial well-being and long-term security [7–10]. However, there is no universally accepted definition or measurement approach for financial literacy [11]. While some scholars conceptualize it primarily as financial knowledge, others emphasize financial attitudes, behaviours, or a combination of these elements [12]. This lack of consensus presents a challenge in understanding the interplay between financial literacy and behavioural biases.

This study aims to bridge this gap by examining the relationship between financial biases and financial literacy within the context of university education. Specifically, we investigate a range of cognitive biases, including overconfidence, gambler’s fallacy, and herd behaviour, which have been widely explored in behavioural finance research but rarely analysed comprehensively in relation to financial literacy. Understanding these biases is particularly relevant in higher education, where fostering financial literacy and self-sufficiency is crucial for developing informed financial consumers and competent professionals [13]. By analysing these dynamics, our research seeks to inform more effective strategies for reducing biases and enhancing financial literacy among university students.

Methodologically, this paper contributes by constructing multiple financial bias indices through the Benefit of the Doubt (BoD) approach within Data Envelopment Analysis (DEA) framework. To the best of our knowledge, no previous study has applied DEA-BoD to develop composite measures of financial biases. Furthermore, this research integrates a two-stage analysis using truncated regression, exploring the relationship between financial literacy and bias indices in a way that allows for greater flexibility and future extensions [14].

In essence, this research enhances our understanding of the relationship between financial literacy and biases, providing practical insights for policymakers, institutions and educators. As financial decision-making continues to evolve in an increasingly complex environment, ongoing research into these behavioural dynamics is essential for refining theoretical models, enhancing financial education strategies, and promoting more rational and informed financial choices.

The remainder of this paper is structured as follows. Section 2 presents the literature review and hypothesis development. Section 3 describes the dataset, variables and model description. Section 4 shows the results of the multivariate analysis and, finally, section 5 outlines the conclusion remarks.

## 2. Literature review and hypothesis development

Financial biases have received increasing attention in both academic research and financial practice. These biases, often described as systematic deviations from rational behaviour, arise from heuristics, emotions and perceptual distortions [15,16]. According to the orthodox economic paradigm, individuals are assumed to make financial choices rationally and efficiently, optimizing utility based on all available information [1]. However, empirical research in behavioural finance has demonstrated that human decision-making is frequently influenced by heuristics, emotions, and cognitive limitations, leading to suboptimal financial behaviours [3,5].

The theory of bounded rationality [2,3] suggests that financial decisions are shaped by cognitive limitations, information asymmetry, and time constraints [4,17]. As a result, individuals simplify complex decision-making processes by relying on mental shortcuts, which often lead to suboptimal financial choices that can reduce financial stability, hinder wealth accumulation, and increase exposure to economic downturns [15,18].

Despite the relevance of financial biases, there is no consensus on their definition and measurement, leading to diverse methodological approaches. While some studies examine their relationship with financial satisfaction [17] or investment willingness [19], others explore their impact on stock market behaviour and home ownership decisions [20,21]. This high diversity of research variables could be related, at least in part, to the paucity of official surveys that take into account qualitative variables about individuals’ perceptions and expectations. Table 1 provides a summary of empirical studies analysing financial biases, highlighting their theoretical frameworks, dependent and independent variables, and key findings.

**Table 1:** Summary of empirical literature based on own surveys analysing financial biases.

Paper	Sample [*Source –* Theoretical framework]: *Period *(place)	Dependent variable (type of measure): *Detail* [*Statistical models*]	Main independent variables (type of measure): *Detail* [Main results]
Adil et al. [15]	253 individual investors [*Own survey –* n.d.] (India)	Quality of investment decisions (1–5) [*Hierarchical regression analysis*]	Behavioural biases (1–5): *Disposition effect* [() male; () female], *herding behaviour* [(-) male; (-) female], *overconfidence* [(+) male; () female] *and risk-aversion* [(-) male; (-) female] Financial literacy (0–1)*: Knowledge about financial interest, risk diversification and inflation* [(+) direct effect; (+)/ () mediator effect]
Anderson et al. [22]	5,814 LinkedIn members [*Own survey* *–* n.d.]: *January 2014* (United States)	Probability that the respondent answered “Yes” standard questions about precautionary savings and retirement planning (0–1) [*Probit/ OLS*]	Overconfidence [(+)] Financial literacy (1–5): *Actual and Perceived score* [(+)]
Armenteros-Ruiz et al. [23]	109 individual investors [*Own survey –* Prospect theory]: *June 2020* (Spain)	Financial biases: *Overconfidence, gambler*’*s fallacy, herd behaviour and domestic bias* [*chi-square tests*]	Economic and financial education: *Knowledge about financial interest, risk diversification and inflation*
Baker et al. [18]	516 individual investors [*Own survey –* Prospect theory]: *2010 – 2015* (India)	Behavioural biases (1–5) [*Regression analysis*]	Financial literacy (1–3*):* *Knowledge about risk and return, compound interest, portfolio diversification and investment management* [() overconfidence; (-) disposition effect; () anchoring; () representativeness; (+) mental accounting; () emotional biases; (-) herding]
Cascão et al. [20]	210 individual investors [*Own survey –* Prospect theory] (Portugal)	Price and localization of households’ investments: *Importance of criterion* [*SEM*]	Cognitive biases: *Herd behaviour* [()], *gambler’s fallacy* [()], *representativeness* [()], *anchoring* [(+)], *overconfidence* [(+)]
Iram et al. [24]	403 women entrepreneurs [*Own survey –* Heuristic theory] (Pakistan)	Investment decision (construct): *Risk, interest payments and return margins* [*SEM*]	Financial biases (constructs): *Anchoring* [()], *overconfidence* [+], *representativeness* [()], *availability* [(+)] Financial literacy (construct): K*nowledge and abilities* [() mediator effect: Anchoring and Representativeness; (-) overconfidence and Availability]
Jain et al. [19]	327 individual investors [*Own survey –* Regret theory/ Prospect theory/ Bounded rationality theory]: *Abril 2021 – June 2021* (India)	Investment intention (1–5) [*PLS – SEM*]	Personality Traits (1–5): *Big Five personality Factor* [(+)] Overconfidence bias (1–5) [(+) direct effect; () indirect effect] Financial literacy: *Awareness about the time value of money and diversification of investments* [(+) direct effect; () indirect effect]
Madaan & Singh [21]	243 individual investors [*Own survey –* Prospect theory/ Bounded rationality theory] (India)	Investment decision-making [*Regression analysis*]	Behavioural Biases: *Herding* [(+)], *overconfidence* [(+)], *disposition* [()] *and anchoring* [()]
Moya-Ponce & Madrazo-Lemaroy [16]	31 undergraduate students from business studies (18–25 years) [*Own survey –* Behavioural and Bounded rationality theories] (Mexico)	Potential behavioural stress points: *Mistrust, uncertainly avoidance and injunctive norms* [*Qualitative analysis*]	Financial beliefs: *Confidence, advice and heuristics for savings*
Rasool & Ullah [25]	300 individual investors [*Own survey –* Prospect theory/ Heuristic theory]: *June 2018* (Pakistan)	Behavioural biases (1–5): *Representativeness, overconfidence, anchoring, gambler’s fallacy...* [*Ordinal regression model*]	Financial literacy score (0–1*):* *Knowledge about financial products, interest compounding, inflation, money illusion and debt management above the average* [(-)]
Sahi et al. [17]	30 individual investors [*Own survey –* n.d.] (India)	Financial investment decision making [*Qualitative analysis*]	Behavioural biases: *Overconfidence, framing effect, anchoring, representativeness...*
Seraj et al. [26]	180 respondents [*Own survey –* n.d.]: *April – June 2022* (Saudi Arabia)	Investment decisions (construct): *Saving, financial products, risk...* [*PLS – SEM*]	Financial literacy (construct): *Knowledge about stock market* [(+)] Overconfidence (construct): *Feel confident about financial decisions* [(+) mediator effect]
Weixiang et al. [27]	450 individual investors [*Own survey –* Prospect theory/ Learning cognitive theory]: *September 2021 – January 2022* (India)	Quality of investment decisions (1–5) [*SEM*]	Behavioural biases (construct; 1–5): *Herd mentality, heuristics, cognitive illusions, and framing thinking* [(-)] Financial literacy (construct; 1–5): *Competency, proficiency and opportunity* [(+)]

*Notes:* Some information were omitted due to it was not available in the papers. (+)/ (-)/ () indicate positive/ negative/ non-significant relationship, respectively. *PLS – SEM* refers to Partial Least Square – Structural Equation Modelling. (0–1) represents dichotomous variables; n.d. indicates not detailed; and (# to #) stands for scalar variables. *Source:* Authors own work.

A fundamental distinction in this literature is between cognitive or emotional biases [23]. Cognitive biases stem from distortions in information processing, leading individuals to misinterpret data or apply flawed reasoning in financial decisions. Conversely, emotional biases arise from affective and psychological responses, often causing impulsive or risk-averse behaviours. Almost all studies in Table 1 include both cognitive and emotional biases to provide a comprehensive understanding of behavioural distortions in financial decision-making [17,20]. This classification is critical, as the strategies for mitigating biases differ depending on their nature. Cognitive biases may be reduced through structured financial education and analytical tools, whereas emotional biases require behavioural interventions focusing on self-regulation and financial attitudes [28].

A key observation from Table 1 is that overconfidence is the most frequently analysed bias, reinforcing its role in excessive self-assurance regarding financial knowledge and decision-making abilities [15,23]. This bias often leads individuals to underestimate risks and ignore external financial advice. Herd behaviour is also widely studied, as it reflects a tendency to follow collective financial decisions rather than conducting independent evaluations [15,29]. Additionally, anchoring bias is commonly analysed, highlighting how individuals tend to rely excessively on initial reference points, even when they are irrelevant or outdated [5,24]. Representativeness bias, which leads individuals to base financial decisions on past experiences or stereotypes rather than objective analysis, is another recurrent theme [23].

In this study, we focus on overconfidence, gambler’s fallacy, and herd behaviour, complemented with self-serving bias and loss aversion, because these biases are consistently reported as especially relevant among young adults and university students, directly influencing saving, debt, and investment choices [23,30–32]. Recent research further highlights that generational profiles matter: younger cohorts such as Gen Z and Millennials are particularly prone to overconfidence and probability misjudgements, while older groups display stronger status quo and confirmation biases [33]. This generational evidence supports our decision to prioritise biases most prevalent in undergraduate populations. At the same time, we recognise that other biases -such as mental accounting, present bias, or status quo bias- are also influential and frequently documented [33–35].

Most studies in Table 1 suggest that financial biases contribute to irrational investment decisions, excessive risk-taking, and insufficient portfolio diversification [18,26]. However, there is no consensus on how to effectively mitigate biases, leading scholars to investigate potential drivers such as financial literacy. While some studies argue that higher financial literacy reduces biases, others find that even financially knowledgeable individuals are susceptible to irrational behaviour [36,37]. Recent evidence by Merter [38] reinforces this view, showing that knowledge gaps limit the translation of financial literacy into better decision-making, meaning that literacy does not automatically ensure rational behaviour. These inconsistencies indicate that a more holistic approach is needed, incorporating not only financial knowledge but also financial behaviours and attitudes to assess how individuals apply their knowledge in real-world settings [10,12].

Recent research suggests that financial literacy -defined as the combination of knowledge, behaviours, and attitudes- is a more robust framework for understanding financial decision-making [7,8]. While financial knowledge reflects the cognitive dimension, financial behaviours capture actual financial practices, and financial attitudes measure individual predispositions toward financial planning and responsibility. Despite that, most existing studies have focused only on financial knowledge, overlooking how behaviours and attitudes influence biases [28].

An additional limitation in prior research is that most studies have examined financial biases in general populations, neglecting specific demographic groups such as university students. Financial education plays a crucial role in shaping long-term financial behaviours, making students a critical population for studying how financial literacy evolves over time [26]. Despite that, as Table 1 shows, most studies fail to examine how formal financial education are related to biases, leaving a gap regarding whether structured learning can help reduce irrational financial decision-making.

This study addresses these gaps in the literature by introducing three key contributions. First, it constructs composite indices of financial biases using DEA-BoD methodology, providing a more comprehensive measure of financial biases rather than isolated indicators. Second, it integrates all three components of financial literacy -knowledge, behaviours and attitudes- to understand their combined role on mitigating biases. Third, it focuses on university students at different academic levels, enabling an examination of whether financial education reduces biases and improves financial decision-making [16,28].

Based on the literature review, we propose three hypotheses.

*H.1: Financial knowledge is negatively related to financial biases*.

Financial knowledge is widely regarded as essential for making informed financial decisions. Individuals with a higher level of financial knowledge are expected to better assess risks, avoid impulsive choices, and rely less on heuristics when making financial decisions [18]. Prior research suggests that understanding key financial concepts, such as inflation, interest rates, and risk diversification, reduces susceptibility to biases like overconfidence and gambler’s fallacy [15]. However, financial knowledge alone may not fully eliminate irrational behaviours, as even knowledgeable individuals can be influenced by emotions and cognitive shortcuts [37]. Despite this, we hypothesize that greater financial knowledge is associated with fewer financial biases.

*H.2: Healthy financial behaviours are negatively related to financial biases*.

Beyond knowledge, financial behaviours play a crucial role in mitigating biases. Individuals who engage in responsible financial practices, such as budgeting, saving, and financial planning, are less likely to make decisions based on intuition or social influence [7,36]. Sound financial habits promote critical thinking and reduce reliance on heuristics, helping individuals to resist herd behaviour and avoid excessive risk-taking. Previous studies indicate that structured financial behaviours act as a safeguard against biases, fostering more rational and informed financial decision-making [15,29]. Accordingly, we propose that individuals with healthier financial behaviours are less prone to financial biases.

*H.3: Positive financial attitudes are negatively related to financial biases*

Financial attitudes, such as long-term planning, self-control, and financial responsibility, influence how individuals approach decision-making. A proactive financial attitude encourages individuals to engage in more deliberate and goal-oriented financial behaviours, reducing susceptibility to biases like framing effects or overconfidence [10,12]. Those with stronger financial attitudes are more likely to resist impulsive decisions and demonstrate greater financial resilience, even in uncertain market conditions. Therefore, we hypothesize that individuals with positive financial attitudes exhibit lower levels of financial biases.

In addition, this study examines whether academic level and gender influence the relationship between financial literacy and biases, exploring whether formal education and gender differences can improve financial decision-making and reduce behavioural distortions [28].

## 3. Methodology

### 3.1. Sample selection and field work

The fieldwork was conducted in September 2023. Questionnaires were distributed to first-year and fourth-year students enrolled in the Business Administration and Economics programs at the University of Santiago de Compostela (USC). At the moment of administering the survey in the classroom, students were informed about the objectives of the study and explicitly asked for their permission to participate. They were told that participation was voluntary and that they could decline to respond if they did not agree. Therefore, the act of completing the questionnaire constitutes the documentation of their acceptance. This procedure was reviewed and approved by the University of Santiago de Compostela Research Ethics Committee (IRB).

Prior to distribution, the questionnaire underwent rigorous testing with the assistance of students whose profiles resembled those of the study’s target audience. This process led to the refinement and finalization of the questionnaire.

To ensure effective fieldwork execution and maintain control over the target audience completing the questionnaires, the involvement of professors from the respective degree levels was solicited. This approach ensured the distribution and collection of questionnaires were conducted in person during class sessions. The professors’ cooperation significantly contributed to the study’s high response rate, with only three students opting not to participate.

The survey was administered exclusively in Spanish, considering all respondents were native Spanish speakers. Four Erasmus program students were excluded from participation. After excluding seven incomplete questionnaires, the final sample consist of 403 university students, with 234 first-year and 169 fourth-year students. Regarding gender, sample is formed by 204 males and 199 females.

The questionnaire comprised two sections. The first explored questions related to behavioural biases influencing investment decisions, specifically overconfidence, gambler’s fallacy/ hot hand fallacy and herd behaviour (Table 2). The second section aimed to assess financial literacy, including variables related to knowledge, behaviours and attitudes (Table 3).

**Table 2:** Indicator synthesis for the first-stage analysis.

Behavioural bias	Initials	Questionnaire questions	Course *First Fourth*	Gender *Male Female*
Over confidence	III_1_INST	Imagine that you have done very well after investing in the stock market. How likely is it that next time you invest in financial products you will be guided by your instincts? *5 (Not likely) to 1 (Very likely)*	**2.662 2.947**	**2.960 2.598**
	III_2_EXPE	What do you consider the appropriate way to gain experience in the stock market? *3 (By experiencing losses) – 2 (By obtaining profits)- 1 (Just by observing the market without participating in it)*	1.872 1.929	1.985 1.804
	III_3_TREN	In which market would you invest more? *2 (It does not matter to me) – 1 (In a bullish market; In a bearish market)*	**1.372 1.225**	1.328 1.291
Gambler’s/ hot hand fallacy	III_4_CONT	The shares of a certain company have increased in value over the last two weeks, what do you think will happen tomorrow? *2 (It is not known) – 1 (The shares will continue to rise; The shares will fall)*	1.769 1.840	1.794 1.804
	III_5_PROBI	If you, due to the investments you have made, have lost three times in a row, in the next move it is more likely that: *2 (You do not know) – 1 (I will beat the market; I will not beat the market)*	**1.697 1.787**	1.731 1.739
	III_6_PROBE	If in the previous lottery draw the winning number was 74,908, what are the chances that the same number will come out in the next draw? *1 (The same likelihood) – 0 (Less likely; More likely)*	1.795 1.858	1.843 1.799
Herd behaviour	III_7_PEOP	In a discussion where most participants believe that the shares of a certain company are going to rise, what would you say if asked? *2* (*I will maintain my opinion) – 1 (My opinion will be influenced)*	**1.517 1.396**	1.456 0.482
	III_8_MAJO	If you share the same opinion as the majority…*2 (I will perceive the same risks) 1 (I will perceive more risks; I will perceive fewer risks)*	1.577 1.556	1.534 1.477
	III_9_CRIS	How would you act in the face of a major stock market crisis? *2 (I would decide to hold; I would buy shares) – 1 (I would sell my shares at any price)*	1.730 1.698	**1.804 1.603**

*Notes:* The values associated with each possible response for each question considered are detailed in italics. The questions and coding criteria have been extracted from Armenteros-Ruiz et al. [23]. 0–1 indicates dummy variable. # to # indicates ordinal variable. The values with significant differences (p < 0.05) are highlighted in bold. *Source:* Authors own work.

**Table 3:** Indicator synthesis for the second-stage analysis. Financial literacy (FL), Gender and Course.

Type	Initials	Questionnaire questions	Course *First Fourth*	Gender *Male Female*
FL. Knowledge	II_A_1_INFL	Imagine you have to wait a year to collect a prize of €1,000 and inflation remains at 5%. In a year’s time you will be able to buy... *2 (Less than I could buy today) – 1 (More than I could buy today; The same amount)*	**1.521 1.834**	**1.751 1.552**
	II_A_4_INTC	Suppose you deposit 100€ into a savings account with a guaranteed annual interest rate of 2% (tax-free). You make no additional deposits and do not withdraw any money. How much will be in the account after five years (remembering that there are no taxes or tax deductions)? *1 (Less than €110) – 2 (€110 or more)*	**1.713 1.934**	**1.887 1.723**
	II_A_5_RISK	If someone offers you the chance to make a lot of money, there is probably also the chance that you will lose a lot of money *1 (False) – 2 (True)*	1.982 1.946	1.955 1.979
FL. Behaviour	II_B_1_PLEX	I carry out regular planning of my spending and set savings targets *1 (Totally disagree) to 5 (Totally agree)*	3.175 3.201	3.258 3.105
	II_B_2_LTOB	I set long-term financial goals and try to achieve them *1 (Totally disagree) to 5 (Totally agree)*	3.004 2.775	2.985 2.814
	II_B_3_ANFS	I have thought of specific strategies to avoid becoming a victim of fraud *1 (Totally disagree) to 5 (Totally agree)*	**2.935 2.639**	2.921 2.698
	II_B_4_PRDA	I am very careful with the protection of my data (account number, passwords, etc.) *1 (Totally disagree) to 5 (Totally agree)*	**4.094 3.775**	3.926 3.995
	II_B_5_COPR	I compare prices and conditions from different suppliers before a purchase decision *1 (Totally disagree) to 5 (Totally agree)*	4.281 4.331	4.240 4.367
	II_B_6_ADCO	I adjust my spending and purchasing decisions to changes in my financial situation *1 (Totally disagree) to 5 (Totally agree)*	4.179 4.177	4.196 4.161
FL. Attitude	II_B_7_LIDA	I tend to live from day to day without worrying too much about the future *1 (Totally agree) to 5 (Totally disagree)*	3.871 3.893	3.852 3.909
	II_B_8_SBTS	I am happier to spend money than to save it in the long run *1 (Totally agree) to 5 (Totally disagree)*	3.786 3.674	3.838 3.638
	II_B_9_WPEX	I take care to pay my regular expenses *1 (Totally disagree) to 5 (Totally agree)*	4.034 4.177	4.044 4.145
Course	I_1A_COUR	What grade are you currently in? *0 (First) – 1 (Fourth): frequencies*	234 169	-- --
Gender	V_2_GEND	Gender *0 (Male) – 1 (Female): frequencies (3 missing values)*	-- --	204 199

*Notes:* The values associated with each possible response for each question considered are detailed in italics. The questions and coding criteria have been extracted from OECD [39]. 0–1 indicates dummy variable. # to # indicates ordinal variable. The values with significant differences (p < 0.05) are highlighted in bold. *Source:* Authors own work.

### 3.2. Measurement of variables

The survey items described in the previous section were operationalised as indicators of behavioural biases and financial literacy. The bias indicators corresponding to each dimension were used as outputs in the DEA-BoD analysis. Measurement results for both first-year and fourth-year students, as well as gender differences, are reported in Table 2.

The overconfidence bias was assessed through questions about reliance on instinct, learning methods in financial markets, and preference for different market conditions. The gambler’s fallacy/ hot hand fallacy was measured by evaluating participants’ perceptions of stock price patterns and their beliefs about future market movements based on past trends. Finally, herd behaviour was analysed through questions on susceptibility to peer influence, perceived risk in collective decision-making, and reactions to market crises. These questions allowed for a multidimensional assessment of financial biases, differentiating between cognitive distortions and emotional responses.

The classification of behavioural bias items into overconfidence, gambler’s fallacy, and herd behaviour is grounded in the behavioural finance literature. Overconfidence has been consistently documented as a determinant of excessive risk-taking and poor diversification [18,30]. Gambler’s fallacy, linked to the representativeness heuristic, captures probability misjudgements frequently observed in financial contexts [5,31]. Herd behaviour represents the social tendency to imitate collective decisions rather than conduct independent evaluations [29,32]. This tripartite classification is also aligned with more recent applied frameworks [17,23]. The variation in responses across academic levels and gender groups provides insights into how formal financial education and individual predispositions shape bias susceptibility.

Additionally, the correlation matrix (Table 4) offers an initial overview of the relationships among components of financial biases -overconfidence, gambler’s fallacy, and herd behaviour-. The correlations are generally low and positive, indicating that the variables capture distinct but conceptually related dimensions of financial bias. This supports the suitability of these indicators for constructing a composite index through the DEA-BoD approach, where each output contributes uniquely to the efficiency score. Notably, the overconfidence dimension, as represented by reliance on instincts (III_1_INST), shows statistically significant correlations with variables linked to herd behaviour, such as majority influence (III_7_PEOP), shared opinions (III_8_MAJO), and crisis-driven reactions (III_9_CRIS), suggesting a behavioural overlap rooted in intuitive and socially influenced decision-making. Similarly, gambler’s fallacy is reflected in the belief that past outcomes predict future gains (III_5_PROBI), which correlates significantly with continued price expectation (III_4_CONT), reinforcing the internal consistency of this dimension. Overall, the structure and empirical relationships among the selected outputs validate their inclusion in the model and support their capacity to reflect complementary behavioural patterns relevant for the composite bias index.

**Table 4:** Correlation Coefficients Matrix for the first stage analysis.

	*III_1_INST*	*III_2_EXPE*	*III_3_TREN*	*III_4_CONT*	*III_5_PROBI*	*III_6_PROBE*	*III_7_PEOP*	*III_8_MAJO*
**III_1_INST**	1							
**III_2_EXPE**	0.0184	1						
**III_3_TREN**	0.0323	−0.0994	1					
**III_4_CONT**	0.0963	−0.0028	0.0749	1				
**III_5_PROBI**	0.0723	−0.0242	−0.0424	**0.3749**	1			
**III_6_PROBE**	−0.0179	−0.0503	−0.0724	**0.0866**	**0.1266**	1		
**III_7_PEOP**	**0.1361**	−0.0329	0.0067	−0.0026	0.0100	−0.0427	1	
**III_8_MAJO**	**0.1330**	−0.0477	−0.0003	0.0857	0.0976	0.0371	0.0602	1
**III_9_CRIS**	**0.1035**	0.0144	0.0416	0.0127	0.0078	0.0810	0.0204	−0.0116

*Note:* In bold 5% significance level. *Source:* Authors own work.

The composite bias indices obtained through the DEA-BoD approach should be interpreted as relative measures of susceptibility rather than absolute scales. A score of 1 corresponds to the efficiency frontier, representing minimal susceptibility to biases, while values greater than 1 indicate increasing vulnerability in comparison with peers in the sample. These indices therefore capture relative positions within the distribution instead of establishing fixed thresholds to define “high” or “low” bias. Their main usefulness lies in enabling meaningful comparisons across groups (e.g., first- versus fourth-year students, or male versus female respondents), thereby highlighting systematic patterns in bias susceptibility.

The exogenous variables used for the second part of our analysis are detailed in Table 3. The indicators employed to assess financial literacy were categorized into three key dimensions: knowledge, behaviours, and attitudes. The financial knowledge dimension was evaluated through three questions, assessing students’ understanding of inflation, interest rates, and risk perception. The results suggest higher financial knowledge among fourth-year students, particularly in concepts of interest rates and inflation, which aligns with expectations given their academic progression. Moreover, risk perception scores were consistently high across all groups, suggesting that students generally recognize the probabilistic nature of financial decisions.

The financial behaviours dimension focused on budgeting, long-term planning, fraud prevention, data security, and consumer comparison habits. The data indicate that fourth-year students score higher in structured financial behaviours, such as planning expenses, comparing prices before purchases, and adjusting financial decisions based on their situation. Notably, fraud awareness and data security measures showed strong responses across all groups, suggesting a widespread understanding of cybersecurity risks in financial transactions.

The financial attitudes dimension captured students’ perspectives on financial prudence, long-term saving, and overall financial well-being. Fourth-year students exhibited a slightly stronger inclination toward structured financial habits, such as saving rather than spending and ensuring timely expense payments. However, gender-based differences highlight that female students generally demonstrate stronger financial planning attitudes, a trend observed in prior research on financial decision-making behaviours.

### 3.3. Empirical strategy

To study the presence of overconfidence, gambler’s/ hot hand fallacy and herd behaviour biases among students and how financial literacy influence the presence of these biases, we used a two-stage methodology. Firstly, we constructed composite indices (CIs) for each of the biases and a global indicator to measure the financial bias of each student. Secondly, we performed truncated regressions with the financial literacy indicators, gender and course as explanatory variables for the different previously estimated indices.

#### 3.3.1. *First stage: Composite Index.*

In the first stage, we employed a nonparametric Data Envelopment Analysis with the Benefit-of-the-Doubt (DEA-BoD) approach to construct CIs of behavioural biases. DEA-BoD is widely used to generate efficiency-based indicators when multiple outputs are involved and no a priori weights are available [40]. In our case, the outputs correspond to the items capturing overconfidence, gambler’s fallacy, and herd behaviour, as described in Section 3.2 (Table 2).

The method assigns optimal weights endogenously to each item for every individual, thereby maximising the relative performance score subject to the constraint that no respondent can exceed the efficiency frontier. This procedure allows us to obtain bias indices that are data-driven, objective, and comparable across individuals. As is standard in DEA, the efficiency frontier is normalised to one, and scores above one indicate greater susceptibility to biases relative to peers. Bootstrap resampling was applied following Simar & Wilson [41] to obtain bias-corrected efficiency estimates and confidence intervals for the indices.

The DEA-BoD model can be formally expressed as follows for a decision making unit (DMU)*j*:


$${CI}_{j}=\begin{array}{c} max\\ \lambda_{i} \end{array}\sum_{i=\text{1}}^{m}\lambda_{i}y_{ij}$$

s.t.: $\sum_{i=\text{1}}^{m}\lambda_{i}y_{ij}\;\leq \text{1}\;j=\text{1},\;\ldots ,\;n$


$$\lambda_{i}\;\geq \text{0}\;i=\text{1},\;\ldots ,\;m$$

where *n* is the number of DMUs (i.e., students), *m* is the number of partial indicators, *CI* is the composite indicator score, and *λ* is the weighting.

#### 3.3.2. *Second stage: Truncated regression.*

In the second stage, we employed Algorithm II from the truncated regression model proposed by Simar and Wilson [42] to assess the explanatory power of specific exogenous variables on the CI. The second-stage regression is formulated as follows:


$${\hat{\hat{CI}}}_{i}=\beta Z_{i}+\varepsilon_{i}\;\;i=\text{1},\;\text{2},\;\ldots ,\;n$$

where ${\hat{\hat{CI}}}_{i}$ indicates the dependent variable, specifically the bootstrapped bias-corrected CI for student_*i*_. Moreover, $Z_{i}$ is a vector of exogenous variables which is expected to explain the CI variations; β is a vector of parameters, which defines the relationship between the independent variables and the CI, to be estimated in the second stage. Finally, $\varepsilon_{i}$ is an independent error term following the normal distribution with a zero mean and $\sigma_{\varepsilon }^{\text{2}}$ variance $N\left(\text{0},\sigma_{\varepsilon }^{\text{2}}\right)$ truncated on the left tail $\;\left(\text{1}-\hat{\beta }Z_{i}\right)$.

This procedure corrects for the serial correlation and finite-sample bias inherent in DEA efficiency scores and provides valid confidence intervals for the estimated coefficients. It represents the benchmark in the DEA literature and overcomes the limitations of conventional approaches such as OLS or Tobit regressions, which yield biased and inconsistent inference when applied to DEA scores.

In this stage, the dependent variables were the bias indices derived from the DEA-BoD analysis (both the global index and the separate indices for overconfidence, gambler’s fallacy, and herd behaviour). The explanatory variables included the three financial literacy dimensions—knowledge, behaviours, and attitudes—along with the socio-demographic controls (academic year and gender). This specification allowed us to assess the influence of financial literacy on susceptibility to behavioural biases while controlling for observable heterogeneity.

To address potential concerns about multicollinearity among explanatory variables, we calculated Variance Inflation Factors (VIF) for all predictors. Since the same set of variables was included across models, VIF values were identical and ranged between 1.03 and 1.55, well below conventional thresholds (5 or 10). This confirms that collinearity is not an issue in our specifications. In addition, we re-estimated reduced models by literacy dimension (knowledge only, behaviour only, and attitude only). The results remained consistent with those of the saturated specification, reinforcing the robustness and stability of our findings.

The DEA-BoD framework combined with the double-bootstrap truncated regression ensures that our results are statistically sound and methodologically consistent with best practices in the efficiency analysis literature. The robustness checks (VIF diagnostics and reduced models) further support the validity of our findings. While the indices should be interpreted as relative rather than absolute measures of susceptibility, the consistency of results across specifications reinforces the reliability of our conclusions.

## 4. Results and discussion

### 4.1. *First stage: construction of the CI*

Using the Benefit of the Doubt (BoD) methodology, we calculated the CIs for overconfidence (OVER), gambler’s fallacy (GAM), and herd behaviour (HERD), as well as the global index (GLOBAL) that captures overall financial bias. Higher values on these indices indicate greater susceptibility to biases or lower levels of rationality, whereas values closer to 1 reflect more rational financial behavior.

The descriptive statistics (Table 5) indicate that overconfidence is the most pronounced bias, followed by herd behaviour and gambler’s fallacy. The global index suggests that financial decision-making among students deviates significantly from rational models, reinforcing the impact of behavioural distortions in financial choices.

**Table 5:** Statistic descriptive of Overconfidence, Gambler and Herd, and Global Indices.

	*OVER*	*GAM*	*HERD*	*GLOBAL*
Mean	1.2321	1.0348	1.0770	1. 2321
*Mean first course*	*1.2181*	***1.0556***	*1.0600*	*1.2181*
*Mean fourth course*	*1.2515*	***1.0060***	*1.1007*	*1.2515*
*Mean male*	***1.1942***	*1.0393*	***1.0442***	***1.1942***
*Mean female*	***1.2710***	*1.0302*	***1.1107***	***1.2709***
Standard deviation	0.3717	0.1833	0.2669	0.3717
Range	1.5005	1.0001	1.0002	1.5005
Minimum value	1.0001	1.0001	1.0000	1.0001
Maximum value	2.5006	2.0002	2.0003	2.5006

*Note:* Values in bold indicate statistically significant differences at the 5% level (p < 0.05), according to independent-samples t-tests. Equality of variances was assessed using Levene’s test. *Source:* Authors own work.

An analysis of the indices by academic year and gender reveals important differences. The results indicate that first-year students exhibit higher levels of gambler’s fallacy compared to fourth-year students, suggesting that exposure to financial education and experience over time may help reduce this bias. However, herd behaviour and overconfidence biases do not show significant improvements with academic progression, implying that knowledge acquisition alone may not be sufficient to counteract deeply ingrained cognitive tendencies.

Gender differences are also evident, with female students displaying higher levels of overconfidence and herd behaviour, while male students exhibit a lower global bias index. The persistence of biases across gender groups emphasizes the need for financial education programs that address behavioural tendencies alongside technical knowledge.

### 4.2. Second stage: Truncated regression

The results of the truncated regression (Table 6) reflect the complex nature of financial decision-making, which extends beyond the orthodox paradigm of perfect rationality described by Fama [1] and Simon [2]. Conversely to the expectations of rational actors maximizing utility, none of the financial knowledge indicators significantly explain the GLOBAL index or the individual biases OVER, GAM, or HERD. These findings, which contrasts with the results of Baker et al. [18], Rasool & Ullah [25] and Seraj et al. [26], underscores the theory of bounded rationality [2,3], suggesting that cognitive constraints and a reliance on heuristics, rather than purely rational calculations, limit effective decision-making.

**Table 6:** Results: Truncated regression.

	*GLOBAL*			*OVER*			*GAM*			*HERD*		
	**beta**	**LI**	**LS**	**beta**	**LI**	**LS**	**beta**	**LI**	**LS**	**beta**	**LI**	**LS**
(Intercept)	0.7395	0.2919	1.3447	2.0320	1.2270	2.8230	1.6206	1.1367	2.1435	1.1781	0.5533	1.8375
II_A_1_INFL	−0.0128	−0.0842	0.0656	−0.0704	−0.2001	0.0642	0.0123	−0.0673	0.0935	0.0470	−0.0543	0.1467
II_A_4_INTC	0.0479	−0.0477	0.1350	0.1074	−0.0367	0.2575	−0.0270	−0.1215	0.0644	0.0255	−0.0948	0.1463
II_A_5_RISK	0.0765	−0.1699	0.2534	0.2443	−0.0950	0.5685	−0.0266	−0.2284	0.1653	0.1028	−0.1784	0.3611
II_B_1_PLEX	0.0062	−0.0289	0.0416	0.0145	−0.0461	0.0741	−0.0121	−0.0507	0.0258	0.0115	−0.0343	0.0590
II_B_2_LTOB	0.0207	−0.0130	0.0527	−0.026	−0.0797	0.0288	0.0122	−0.0212	0.0440	**0.0577**	**0.0156**	**0.1002**
II_B_3_ANFS	0.0203	−0.0055	0.0475	0.0264	−0.0210	0.0736	0.0088	−0.0191	0.0376	**0.0321**	**0.0051**	**0.0682**
II_B_4_PRDA	−0.0128	−0.0466	0.0196	−0.0300	−0.0855	0.0295	−0.0095	−0.0467	0.0273	0.0198	−0.0260	0.0652
II_B_5_COPR	**0.0348**	**0.0053**	**0.0764**	0.0405	−0.0353	0.1118	**0.0619**	**0.0171**	**0.1051**	−0.0148	−0.0721	0.0419
II_B_6_ADCO	−0.0168	−0.0581	0.0207	−0.0265	−0.0902	0.0431	−0.0097	−0.0505	0.0310	−0.0211	−0.0711	0.0306
II_B_7_LIDA	**0.0406**	**0.0017**	**0.0754**	**0.0598**	**0.0022**	**0.1191**	0.0265	−0.0103	0.0621	**0.0651**	**0.0153**	**0.1124**
II_B_8_SBTS	0.0124	−0.0204	0.0459	0.0114	−0.0417	0.0676	−0.0024	−0.0352	0.0320	0.0166	−0.0318	0.0616
II_B_9_WPEX	**−0.0368**	**−0.0692**	**−0.0035**	**−0.0632**	**−0.1222**	**−0.0015**	−0.0090	−0.0453	0.0280	−0.0350	−0.0853	0.0170
I_1A_COUR	0.0046	−0.0204	0.0294	0.0070	−0.0361	0.0485	**0.0294**	**0.0040**	**0.0545**	**−0.0384**	**−0.0725**	**−0.0037**
V_2_GEND	**−0.0555**	**−0.1202**	**−0.0122**	**−0.2350**	**−0.3482**	**−0.1273**	−0.0073	−0.0827	0.0642	0.0340	−0.0579	0.1258
Sigma	2.0672	1.4418	3.6267	0.6770	0.6424	0.7341	1.6206	1.1367	2.1435	0.4259	0.3985	0.4699

*Note:* Significant coefficients, for a 95% confidence level, are shown in bold. *Source:* Authors own work.

The analysis reveals notable relationships between financial behaviours and biases. Individuals who frequently compare financial options before making purchasing decisions (COPR) tend to exhibit stronger probability of misjudgement biases (GLOBAL and GAM). This behaviour suggests that their decision-making may be shaped more by past experiences and perceived patterns than by independent evaluation. This reliance on external cues aligns with previous findings [25,31,43], which demonstrate how individuals incorporate the experiences of others into their decision processes, reinforcing heuristic-driven biases. The observed association between comparative decision-making and increased susceptibility to probability misjudgement indicates that individuals may simplify complex financial decisions through heuristics, which can lead to systematic errors [5].

Group influence is particularly prevalent among individuals who set long-term financial goals (LTOB) and those who actively develop fraud-avoidance strategies (ANFS). This implies that, rather than relying exclusively on independent analysis, these individuals may conform to group norms or external recommendations as a strategy for navigating uncertainty. The influence of social factors and emotional considerations in financial decisions supports Keynes’ [6] argument regarding the role of animal spirits in shaping economic choices. Additionally, as Spyrou [32] notes, group influence can emerge in response to uncertainty and limited information, prompting individuals to align their financial decisions with prevailing market trends.

Attitudinal variables also play a critical role in explaining biases. A short-term financial perspective (LIDA), characterized by a lack of concern for future financial stability, significantly increases confidence and group influence biases (GLOBAL, OVER and HERD). The findings indicate that individuals who focus on immediate financial needs rather than long-term planning are more prone to emotional and heuristic-driven decision-making. Conversely, maintaining structured financial habits, such as ensuring regular payment of financial obligations (WPEX), is associated with more rational financial decision-making and reduced bias levels (GLOBAL and OVER). In particular, consistently managing financial responsibilities appears to mitigate both overconfidence and general bias levels, underscoring the importance of disciplined financial habits.

Gender disparities further illustrate behavioural tendencies in financial decision-making. Male respondents exhibit higher overall and confidence-related biases, reflecting the well-documented tendency for men to overestimate their financial knowledge and take greater risks [15,44]. These findings suggest that financial education should incorporate behavioural insights to address gender-based differences in financial decision-making.

Academic progression appears to have a limited role on bias reduction. While students in more advanced stages of their studies exhibit lower levels of group influence bias, other biases remain unchanged or even increase (GAM). This challenges the effectiveness of financial education in mitigating cognitive biases and reinforces previous research questioning the long-term role of financial literacy programs [7,45]. The persistence of biases despite academic exposure suggests that financial education alone may not be sufficient to overcome ingrained decision-making patterns.

As a robustness check, we also estimated reduced models separately for each dimension of financial literacy: first using only knowledge variables, then only behavioural variables, and finally attitudinal variables. Although these additional estimations are not shown in Table 6, their results confirmed the main relationships identified in the full specification, reinforcing the robustness and stability of our findings. These additional results are available from the authors upon request.

### 4.3. Discussion

This study examined how the knowledge, behaviour, and attitude dimensions of financial literacy shape the presence of cognitive biases among undergraduate students. The results provide several important insights into the complexity of financial decision-making in young adults.

First, our analysis shows that financial knowledge, while essential in traditional approaches to financial literacy, does not play a decisive role in reducing biases such as overconfidence, gambler’s fallacy, or herd behaviour. Recent evidence reinforces this point: Merter [38] shows that knowledge gaps constrain the ability of financial literacy to improve decision-making, indicating that even financially knowledgeable individuals remain susceptible to heuristic-driven errors. Similarly, previous studies have argued that literacy does not always prevent irrational tendencies [37,45]. This finding implies that H.1 is not supported, as financial knowledge did not significantly reduce bias levels in our models.

Second, attitudinal factors emerge as the most consistent protective element against biases. Students who display stronger attitudes of responsibility, prudence, and financial discipline are less likely to rely on heuristic-driven judgments. This finding underscores the importance of including attitudinal training -such as fostering long-term responsibility and resilience- within financial education programs [7,10]. Accordingly, H.3 is strongly supported, confirming that attitudes are the most reliable dimension for bias mitigation.

Third, our results highlight a paradoxical but highly relevant outcome: behaviours that are typically regarded as markers of financial capability, including long-term planning and fraud avoidance, are associated with greater susceptibility to biases like gambler’s fallacy and herd behaviour. This counterintuitive finding suggests that, in the context of undergraduate students, such behaviours may not always reflect deliberate and rational strategies. Instead, they may function as defensive or risk-averse responses to uncertainty, leading individuals to rely excessively on rules-of-thumb or social imitation [6,31,32]. In this sense, planning and fraud avoidance may act less as indicators of competence and more as coping strategies shaped by limited experience and strong contextual influences. Recognizing this complexity enriches the interpretation of financial behaviours and calls for a more accurate understanding of financial capability in younger populations. Thus, H.2 receives only partial support: while some responsible practices may reduce bias, others paradoxically reinforce it.

Fourth, gender and academic progression appear to influence the manifestation of biases in distinct ways. Male students tend to exhibit stronger confidence-related, and global biases. In addition, although fourth-year students show some reduction in gambler’s fallacy, other biases remain largely unchanged across academic levels. These findings are consistent with prior research documenting gender differences in financial confidence and decision-making [15,44]. They also suggest that formal education, while beneficial in some respects, may not be sufficient to counteract deeply ingrained behavioural tendencies.

Taken together, these findings demonstrate that financial literacy is not a uniform shield against biases but a multidimensional construct with uneven effects. Knowledge, behaviours, and attitudes interact with cognitive and social mechanisms in ways that can both mitigate and exacerbate irrational tendencies. This reinforces the need for financial education policies that move beyond the transfer of theoretical knowledge and place greater emphasis on applied learning, emotional awareness, and behavioural adaptability. Programs that simulate real-world decision-making scenarios and incorporate behavioural insights are more likely to prepare students for the complexities of financial life [46,47].

## 5. Conclusions

This paper analysed the relationship between financial literacy and behavioural biases among undergraduate students, introducing a novel methodological contribution through the use of DEA-BoD to construct composite indices of overconfidence, gambler’s fallacy, and herd behaviour, complemented with a double-bootstrap truncated regression. This approach allowed for a multidimensional and robust assessment of how literacy components—knowledge, behaviours, and attitudes—shape bias susceptibility.

The findings reveal several important contributions and complex interactions between financial literacy and biases. First, financial knowledge does not significantly reduce biases, confirming that literacy based purely on information is insufficient to prevent heuristic-driven distortions. Second, attitudinal factors emerge as more effective than knowledge in mitigating biases, highlighting the importance of financial responsibility and prudence in fostering rational financial behaviour. Third, the study uncovers a paradoxical but highly relevant result: behaviours usually regarded as markers of financial capability—such as long-term planning and fraud avoidance—may, in the context of undergraduates, increase susceptibility to biases. This suggests that these behaviours, at early stages of financial maturity, can act as defensive or risk-averse strategies rather than indicators of competence, opening new avenues for research on how young adults internalize financial practices. In addition, gender differences influence bias patterns, with men showing greater confidence-related biases.

At the theoretical level, this study challenges existing assumptions regarding the link between financial literacy and biases, suggesting that this relationship may vary among young university students compared to the general population. From a practical standpoint, the findings suggest that financial education programs should not only deliver theoretical content necessary for optimal financial decision-making, but also promote experiential and applied learning. It is essential to design formative interventions that prepare students to responsibly manage their finances from the moment they begin to handle personal wealth. Institutions and educators should tailor these interventions to address specific biases, taking into account the differentiated role of knowledge, behaviours and attitudes on financial biases.

Naturally, it is essential to acknowledge the study’s limitations. The sample is restricted to one university, limiting external validity; the cross-sectional design prevents causal inference; the reliance on self-reported data may capture intentions rather than actual behaviour; and the absence of qualitative or experimental methods reduces the ability to identify underlying mechanisms. Moreover, the analysis focused on three major biases, but other distortions such as mental accounting, present bias, anchoring, or status quo bias were not considered. As behavioural economics suggests, biases do not operate in isolation, and future research should examine how different distortions interact or overlap. Expanding to more diverse populations, employing longitudinal or experimental designs, and combining survey data with behavioural tasks and qualitative insights would further strengthen the evidence base.

In sum, this study demonstrates that attitudes -more than knowledge or behaviours- play a decisive role in mitigating financial biases. By highlighting both protective and paradoxical effects of literacy components, it advances theoretical understanding and offers practical guidance for designing financial education programs that are more comprehensive, bias-sensitive, and effective in shaping rational decision-making.

### Declaration of generative AI and AI-assisted technologies in the writing process

During the preparation of this work the authors used DeepL and ChatGPT in order to improve language and readability. After using this tool, the authors reviewed and edited the content as needed and take full responsibility for the content of the publication.
